# Toxic Effects of Copper Nanoparticles on *Paramecium bursaria*–*Chlorella* Symbiotic System

**DOI:** 10.3389/fmicb.2022.834208

**Published:** 2022-03-23

**Authors:** Bingyu Tan, Yiwen Wang, Zhiwei Gong, Xinpeng Fan, Bing Ni

**Affiliations:** ^1^School of Life Sciences, East China Normal University, Shanghai, China; ^2^School of Physics and Electronic Science, East China Normal University, Shanghai, China

**Keywords:** copper nanoparticles, *Paramecium bursaria*, symbiotic system, oxidative stress, energy metabolism disordered

## Abstract

Although many reports have demonstrated that nanoparticles can have a negative effect on aquatic organisms, the toxic effects on symbiotic organisms remain poorly understood. The present study conducts ultrastructure, enzyme activity, and transcriptomics to assess the toxic effects to the *Paramecium bursaria*–*Chlorella* symbiotic system from exposure to copper nanoparticles (CuNPs) for 24 h. We found that in both the host and symbiotic algae, CuNP exposure induced high reactive oxygen species level, which leads to oxidative damage and energy metabolism disorder. Moreover, transmission electron micrographs (TEMs) showed that the symbiotic algae in the cytoplasm of *P. bursaria* were enveloped in the digestive vacuole and digested, and the level of acid phosphatase activity increased significantly within 24 h, which indicated that the stability of the symbiotic system was affected after CuNP exposure. We speculated that the increased energy demand in the host and symbiotic algae resulted from oxidative stress, precipitating the decrease of the photosynthetic products provided to the host, the digestion of the symbiont, and the destruction of the stable symbiotic relationship. The study provides the first insight into the mechanisms of nanoparticles’ toxicity to the symbiotic relationship in the ecosystem, which may help to understand the environmental effects and toxicological mechanisms of nanoparticles.

## Introduction

Nanoparticles (NPs) have increased dramatically in production and use due to their application in agriculture, biomedicine, the environment, electronics and the textile industry, etc. (Blinova et al., 2010). The widespread use of NPs has largely been due to their particular properties that differ from conventional materials in their catalytic properties, conductivity, and optical and transportation properties (Borm et al., 2006). These properties may cause high interactivity with organisms when NPs are released into the environment, especially in natural aquatic ecosystems. The toxicity of NPs has been reported in many aquatic organisms, and in addition to direct effects on organisms, NPs can be answerable for indirect effects through their dissolution and the liberation of their constituent element (Navarro et al., 2008). Currently, the most widely accepted mechanisms of NP toxicity are oxidative stress and inflammation (Li et al., 2008).

Copper nanoparticles (CuNPs) can release metal species in a controlled manner and can ultimately slow or even inhibit the growth of microorganisms. Thus, they have been mixed or coated in a variety of materials to achieve antibacterial application (Cioffi et al., 2005), and because of this, it also has biological toxicity (Manusadianas et al., 2012; Song et al., 2015; Ostaszewska et al., 2016). In recent years, the toxicity of CuNPs has been demonstrated in many organisms, including arthropods (*Artemia salina*; Cimen et al., 2020), water fleas (*Daphnia* spp.; Song et al., 2015), barnacle larvae (Yang and Wang, 2019), chlorella (*Chlorella sorokiniana*; Barreto et al., 2019), ciliates (*Euplotes aediculatus*; Zhao et al., 2022), and microorganisms of the intestine microbiota of broiler chickens (Sizentsov et al., 2018). According to the results of the abovementioned research, CuNPs are more toxic to small organisms than most other NPs and can produce toxic effects on plankton at concentrations as low as 10^−1^–10^−3^ mg/L (Song et al., 2015; Garncarek et al., 2019). There is no doubt that toxic effects in organisms such as plankton, which are the keystone of the food chain, have the possibility of biomagnification in the food chain (EPA, 2007). At the same time, the imbalance of plankton is likely to affect the stability of the aquatic ecosystem. CuNPs are widely used, but it is very important to evaluate their toxicity and to monitor their potential risks to ecosystems.

What we know about the effect of NPs on organisms is largely based on organisms that exist independently in nature, whereas there are many organisms that survive by forming symbionts with other organisms, which is called symbiosis. Endosymbiosis occurs when one organism grows inside another. The endosymbiosis theory provided an excellent explanation as to the origin of eukaryotes, such as mitochondria and chloroplasts, and it has been widely accepted. Endosymbiosis is still a common phenomenon in nature, which provides favorable experiment materials for the study of horizontal gene transfer and coevolution (He et al., 2019). Most symbiotic relationships are mutualistic, and the biotrophic transportation of host and symbiont has been observed, which is beneficial for the survival of both (Masakazu et al., 2016). It has been demonstrated that the establishment of a symbiotic system benefits organism growth and promotes the host’s survival under extreme conditions (Heike et al., 2011; Pelletreau et al., 2014). Therefore, it is important for the balance of the ecosystem to maintain the normal symbiotic relationship between organisms. However, the toxic effects of NPs on the symbiotic system are still poorly understood.

As one of the model organisms of ciliates, *Paramecium bursaria* harbors hundreds of endosymbiotic *Chlorella* in the cytoplasm and forms a long-term stable endosymbiotic system with the latter. Therefore, *P. bursaria* and their symbiotic algae are used as model organisms to examine endosymbiosis. As previous studies reported, the symbiosis algae are surrounded by a perialgal vacuole (PV) membrane beneath the cell cortex, which prevents the algae from being fused with the host’s lysosome (Fujishima and Kodama, 2012; Kodama and Fujishima, 2013). The *P. bursaria* cells take up algae into the cytoplasm by phagocytosis. The algae are then enveloped by a digestive vacuole (DV) membrane. Subsequently, single green algae bud off from the DV membrane and the undigested algae wrapped by the PV membrane. A relatively stable symbiotic relationship is then formed. The symbiotic relationship of *P. bursaria* and symbiotic algae has been regarded as a mutualistic association because they benefit each other by living together, which is the significance of the establishment of endosymbiosis in *P. bursaria*. Besides, *P. bursaria* and symbiotic algae also retain the ability to grow independently. Both cells can be cultured discretely, whereas re-endosymbiosis can be easily established by mixing isolated algae and algae-free *P. bursaria* (Kodama and Fujishima, 2005). Therefore, the cell and genome of *P. bursaria* and the symbiotic algae in the symbiotic system have been relatively intact, which is a suitable model to study the toxicity of NPs to symbiosis.

Based on the foregoing, the *P. bursaria*–*Chlorella* symbiotic system was used as the testing organism in this study to (i) investigate the toxic effect and mechanisms of CuNPs and (ii) address the effect of nanomaterials on the symbiotic system. Collectively, our findings will provide new directions for exploring the environmental effects and toxicological mechanisms of nanoparticles.

## Materials and Methods

### Culture of *Paramecium bursaria*

*Paramecium bursaria* was collected from Zhongshan Park, Qingdao, China. The collected protozoa are grown in wheat fermentation and feed on the bacteria fermented from wheat grains. The ciliates were then cultured at a 16:8 h light-dark photoperiod in cultured bottles at 25 ± 1°C, and they were cultured to the stationary growth phase before they were transferred to a new culture medium for toxicity tests. This experiment with *P. bursaria* was performed in the same media and conditions.

### Nanoparticle Dispersion and Characterization

The uncoated CuNPs were purchased from Shanghai Macklin Biochemical Co., Ltd. and stored at 4°C. Ten milligram of Cu nanopowder was mixed with 100 ml of ultrapure water to yield a 100 mg L^−1^ stock suspension. Stock suspension of CuNPs was sonicated (60 W, ShangHai ShengYan Ultrasonic Equipment Co., Ltd.) for 30 min before use. Characterization of NP dispersions in the exposure medium was performed at 5 × 10^−2^ mg L^−1^. Transmission electron microscopy (HT7700, Hitachi High-Technologies Corporation, Japan) was used to characterize the size and shape of the CuNPs. The samples were prepared by placing a droplet of the CuNPs on a carbon-coated Cu grid followed by obtaining images.

### Acute Toxicity Tests for Determining 24 h-EC_50_ and 24 h-LC_50_

The toxic exposure experiments were conducted in wet boxes with a concave dish at 25°C. *Paramecium bursaria* cells were inoculated into a series of Cu solutions of different concentrations (1, 0.5, 0.25 0.1, 0.025, and 0.01 mg L^−1^) for 24 h (which is about one reproductive cycle for *P. bursaria*) as a preliminary test. Each treatment was randomly assigned to six replicates, and each replicate was stocked with 20 *P. bursaria* cells in a 500 μl of CuNP solution. The control group was formed in which no NPs were applied, and the optimum living environment of the experimental organism had been provided. Mortality was measured under a dissecting microscope (10–40 X). According to the preliminary experimental results, more detailed concentration gradient nano solutions were adjusted for the determination of LC_50_ and EC_50_, respectively. The data of six replicates were averaged for results analyzed, and half-maximal inhibitory concentration (LC_50_) and concentration for 50% of maximal effect (EC_50_) were calculated.

### Microscopic Analysis

To confirm the cytological damages, scanning electron microscope (SEM; Hitachi, Japan) and transmission electron micrograph (TEM; Hitachi, Japan) analyses were performed. *Paramecium bursaria* cells treated with 5 × 10^−2^ mg/L^−1^ of CuNPs for 24 h in ultrapure water treated as control. For the SEM study, cells were fixed in a 1:6 mixture of 1% O_S_O_4_ and a saturated solution of HgCl_2_ at 4°C for 10 min. Then, the cells were rinsed with 0.1 M of phosphate buffer (PB), dehydrated in a graded series of ethanol, dried with a critical point dryer (Leica CPD300), and coated with gold in an ion coater (Leica ACE600). Observations were performed using a scanning electron microscope at an accelerating voltage of 10 kV (Li et al., 2017). The TEM samples were prepared according to the method described by Gu et al. (2002). Ciliates were prefixed in a 1:1 mixture of 2% O_S_O_4_ and 2.5% glutaraldehyde at 4°C for 10 min. The fixed cells were washed with 0.1 M of PB and then postfixed in 1% O_S_O_4_ at 4°C for 1 h. The postfixed cells were washed again and then dehydrated through a graded series of ethanol and acetones and embedded with Epon812. Ultrathin sections were stained with uranyl acetate and lead citrate and observed with a transmission electron microscope at an accelerating voltage of 100 kV.

### Dual RNA-Seq Analysis

#### Nanoparticle Exposure, RNA Isolation, and Illumina Sequencing

To further explore the toxicity mechanism of CuNPs on *P. bursaria*, we conducted transcriptomic analysis after 24 h of CuNPs treatment, and 0 h was a time control for comparison. Each treatment was conducted in triplicate cultures. A CuNP exposure concentration of 5 × 10^−2^ mg L^−1^, which has been proven to significantly affect the survival and cellular structure damage of *P. bursaria*, was used for exposure treatment with about 5 × 10^4^ cells in each culture. After NP exposure, samples from each replicate were centrifuged and washed with doubly distilled water. Thereafter, *P. bursaria* were collected and snap-frozen in liquid nitrogen and stored at −80°C, and the total RNA was isolated with TRIzol reagent (Invitrogen, United States). The concentration, integrity, and RNA integrity number (RIN) of the total RNA were measured with NanoDrop 2000 (Thermo Scientific, United States), agarose gel electrophoresis, and biological analyzer (Agilent Technologies, United States), respectively. Total RNA (1 μg) with a concentration of more than 450 ng/μl^−1^ and OD 260/280 between 2.0 and 2.2 was used for the library construction at Majorbio (Shanghai, China). mRNA was obtained through purifying total RNA and was broken into fragments of approximately 300 bp. All fragments were reverse transcribed to stable double-stranded cDNA with random hexamers added and end repairing. After library enrichment, a paired-end 150 bp sequencing strategy was performed with Illumina Novaseq 6000.

#### Bioinformatic Analysis

Raw reads for RNA sequencing were conducted using SeqPrep and Sickle to trim adapter sequences, remove poly-N-containing reads, and filter low-quality reads. The Q30 (percentage of the Phred quality score >30) was measured to determine the quality of the sequenced RNA. Alignment to the reference genomes of *P. bursaria* [KM2_mac_JV3_6] and *Chlorella variabilis* [GCF_000147415.1] was performed using TopHat. Transcriptome assembly was performed using Trinity. The assemblies were blasted using six databases: Non-Redundant Protein (NR), Swiss-Prot, Pfam, Clusters of Orthologous Groups of Proteins (COG), Gene Ontology (GO), and Kyoto Encyclopedia of Genes and Genomes (KEGG) for functional annotation. The threshold value was set at *E* value <e^−3^. Differentially expressed genes (DEGs) were estimated using DESeq2. The false discovery rate (FDR) and expression fold change (FC) of genes were used to determine DEGs. Genes showing *p*-adjust <0.05 and |log2FC| ≥ 1 at comparison groups were defined as DEGs. Goatools and Python were used to conduct GO and KEGG enrichment analysis. The threshold value of the GO and KEGG enrichments was set as *p*-adjust <0.05.

#### Validation of RNA Sequencing

To validate the reliability of RNA-Seq, nine genes were randomly selected from the list of DEGs of *P. bursaria* and *C. variabilis*. The design of the primer was based on sequence obtained from the RNA-Seq. RNA samples were used for reverse transcription to cDNA using PrimeScript™ RT Master Mix (TaKaRa Biotech Co., Ltd., Dalian, China). From these cDNA samples, 2 μl from each sample was used in qPCR with a TB Green™ Premix Ex Taq™ II (TaKaRa, China). All reactions were performed in triplicate. All quantifications were performed with 18S rRNA as a reference for normalization, and the relative expression level was calculated with the 2^−△△Ct^ method (Schmittgen, 2008).

### ROS Levels

DHE fluorescent probe was applied to detect the production of reactive oxygen species (ROS) in the endosymbiotic system. Then, 3.0 mg of DHE powder (Beyotime Co., China) was mixed with 300 μl of DMSO and diluted with 0.1 M of PB into 10 μg ml^−1^ stock solutions. The final concentration was adjusted through preliminary experiments. The exposure concentration of 5 × 10^−2^ and 1.5 × 10^−2^ mg L^−1^, which have been measured as 24 h LC_50_ and 24 h EC_50_, were used for exposure treatment with 12 and 24 h. The control group was formed in which no NPs were applied. Next, 7 μl of *P. bursaria* cell suspension was added into 7 μl of fluorescent dye solution on the micro slide and placed at 37°C for 3 min (at dark). Photos were taken with a fluorescence microscope (ZEISS Imager Z.2, Carl Zeiss AG, Germany) and an exposure time of 1 s, and the mean fluorescence intensity was measured using ImagePro Plus.

### Enzyme Activity and Lipid Peroxidation

For the exposure experiments, about 3 × 10^4^ cells were added to the medium, which was preequilibrated with 5 × 10^−2^ mg L^−1^ CuNPs. After treatment for 0, 2, 4, 8, 12, and 24 h, *P. bursaria* was collected by centrifugation. The total proteins, lipid peroxide content (LPO), superoxide dismutase (SOD) activity, catalase (CAT) activity, and acid phosphatase (ACP) activity of the *P. bursaria*–*Chlorella* symbiotic system were determined using commercial detection kits (Nanjing Jiancheng Bioengineering Institute, Nanjing, China). Experiments were performed according to the manufacturers’ instructions. Measurements were obtained using a microplate spectrophotometer (BioTek Instruments Inc., United States), and all experiments comprised three replicates for each group of samples.

### Statistical Analysis

All experiments were conducted in triplicates. Mean SDs, correlation, and regression parameters were calculated using MS-Excel (office 2019). The statistically significant difference between control and treatment was analyzed using one-way ANOVA with the help of SPSS. A value of *p* < 0.05 was accepted as significantly different.

## Results

### Characterization of CuNPs and Toxicity Test

**Figure 1:**
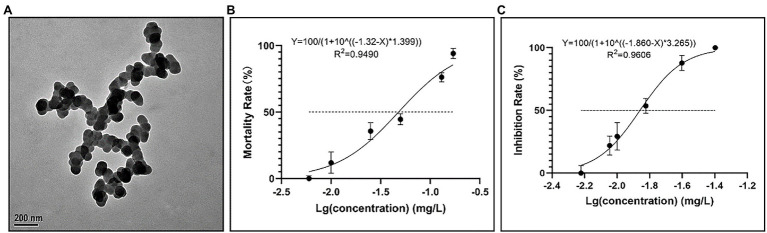
Characterization of copper nanoparticles (CuNPs) and fitting curve. **(A)** Transmission electron micrograph (TEM) of CuNPs form. **(B)** The fitting curve of CuNP toxicity to the mortality of *Paramecium bursaria*. **(C)** The fitting curve of CuNP toxicity to the inhibition rate of *P. bursaria*.

As shown in Figure 1A, the initial TEM measurement showed that CuNPs were spherical particles with an average size of 80 ± 20 nm (Figure 1A). The mortality rate and inhibition rate of *P. bursaria* after 24 h of exposure to different concentrations of CuNPs are shown in Figures 1B,C. The toxicity of CuNPs increased in a concentration-dependent manner. When the concentration of CuNPs reached 0.17 mg L^−1^, *P. bursaria* could hardly survive. Based on the mortality rate, the 24 h LC_50_ was calculated to be 0.04782 ± 0.0056 mg L^−1^ (Figure 1B). Meanwhile, the 24 h EC_50_ of CuNPs was calculated to be 0.0138 ± 0.0007 mg L^−1^ (Figure 1C).

### Microscopic Analysis

**Figure 2:**
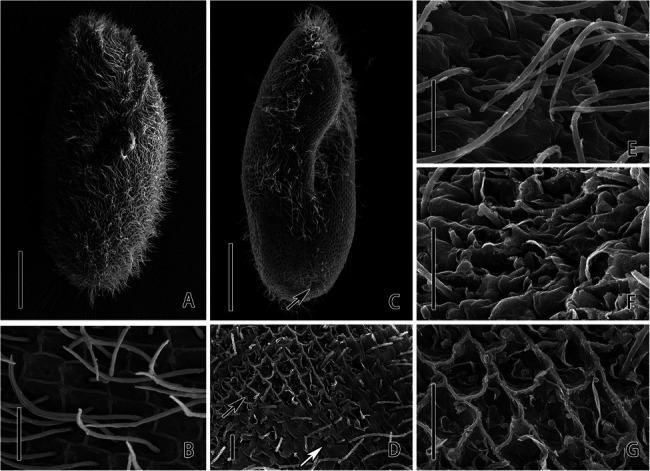
Scanning electron microscopic observation of *P. bursaria* under control **(A,B)** and CuNPs treatment **(C–G)**. **(A)** The cell of *P. bursaria* under control. **(B)** The grids on the surface of *P. bursaria* and a cilium stretch out from the pellicle on the center of the grid. **(C)** The cell of *P. bursaria* under CuNP treatment. The arrow denotes the sunken posterior end. **(D)** The damage on the cortical surface of the cell. The black arrow and white arrow showing the different degrees of damage on the cell. **(E,F)** Three different degrees of surface damage, from slight to severe. Scale bars = 20 μm **(A,C)** and 2 μm **(B,D–G)**.

SEM analysis revealed the detrimental effects to the *P. bursaria* after the exposure of CuNPs. Cell membrane and cilia damage are observed from the micrographs. Images showed that the control cell is long and elliptical in outline with an anterior that is slightly sharp, a posterior end that is broadly rounded, and a body that is densely ciliated (Figure 2A). On the whole surface of *P. bursaria*, there are grids formed by the convex of the pellicle (Figure 2B). In the center of each grid, there is a cilium stretch out from the investigated pellicle. After 24 h of exposure to CuNPs, the cilia of the cell were obviously shed, the posterior end of the cell was sunken (Figure 2C), and the shapes of the grids on the cortical surface were damaged to varying degrees (Figure 2D). The grid depression is deepened in the slightly damaged area (Figure 2E); the cell surface was uneven, and the grid structure was fuzzy (Figure 2F); and the cilia were dropped off in the severely damaged area, and the pellicle structure was damaged (Figure 2G).

**Figure 3:**
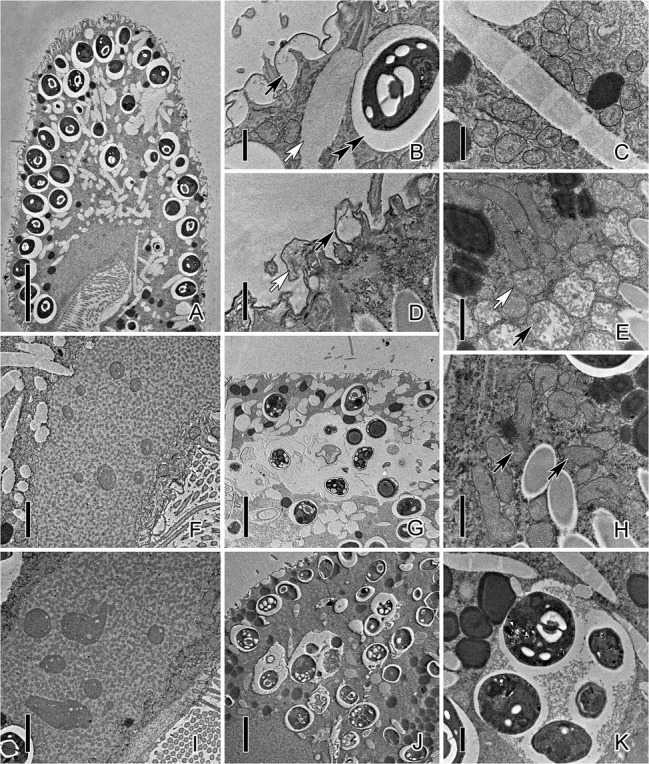
Transmission electron micrograph images of *P. bursaria* under control and CuNP treatment. **(A)** The cell of *P. bursaria* under control. **(B)** The pellicular alveoli (black arrow), symbiotic algae, the PV membrane (double-arrowhead), and trichocyst (white arrow) in untreated *P. bursaria*. **(C)** The mitochondria of the control cell. **(D)** Granular substances (white arrow) or vesicular structures (black arrow) appeared in the pellicular alveoli of CuNP-treated cells. **(E)** Mitochondria were damaged to different degrees. The black arrow showed mitochondria obviously swollen, and the white arrow showed slight swelling. **(F)** The macronucleus of the untreated cell. **(H)** Arrows denote that the membranes of mitochondria were incomplete after treatment. **(I)** The macronucleus of CuNP treatment cell with nucleoli increased. **(G,J,K)** The symbiotic algae were digested in the DV of *P. bursaria* after treatment. Scale bars = 10 μm **(A)**, 5 μm **(G,J)**, 2 μm **(F,I)**, and 1 μm **(B–E,H,K)**.

TEM observation showed significant changes in the inner structure of cells after exposure to CuNPs. The pellicle of control *P. bursaria* was composed of the plasma membrane and the continuous pellicular alveoli (Figure 3A). The symbiotic algae were docking beneath the cortex and were enveloped by a PV membrane, which prevents the algae from being fused by the host’s lysosome. There are many extrusive organelles named trichocysts that usually occupied the position of symbiotic algae (Figure 3B). The mitochondria of the control cell were round or elliptical and composed of a double-layer membrane, with the inner membrane folded inward into cristae (Figure 3C). The macronucleus was elliptical, and nucleoli and chromatin were distributed in the macronucleus (Figure 3F). After 24 h of exposure to CuNPs, mitochondria were seriously damaged. Mitochondria near the pellicle was obviously swollen, and the cristae were fractured and disappeared. Most of the mitochondria in the cytoplasm were deformed or slightly swollen, and some mitochondrial membranes were incomplete (Figures 3E,H). Nucleoli in the macronucleus were prominently increased (Figure 3I). Granular substances or vesicular structures appeared in the pellicular alveoli with serious deformation (Figure 3D). Moreover, the symbiotic algae in the cytoplasm were enveloped in the DV and were digested (Figures 3G,J,K). Because several algae were wrapped in the same vacuole, and other forms of substances were appeared, the vacuole can be considered as DV rather than PV.

Compared with untreated *P. bursaria*, the results of electron micrographs suggested that CuNP exposure significantly changed the structures of *P. bursaria* cells and destroyed the balance of the endosymbiosis system. To further detect the mechanism of CuNP toxicity on *P. bursaria*, dual RNA-seq analyses were performed.

### Transcriptomic Responses

#### Transcriptome Sequencing and Assembly

Overall, more than 10.04 Gb of clean data were obtained in each group, and Q30 was more than 94.03% in the control and CuNP-treated groups. Clean reads were obtained by removing reads containing adapter, undermined base, and low-quality data from raw reads. Clean reads were mapped to a reference transcriptome of *P. bursaria* (KM2_mac_JV3_6), following *de novo* transcript assembly, post-optimization and BLAST, 30028 expressed genes and 43,229 expressed transcripts were obtained. These were functionally annotated using six major databases. The same methods were used to analyze the data of the symbiotic algae *C. variabilis*. The correlation of the biological replicates was shown in Supplementary Figure S1. Raw sequence data associated with this project has been deposited at sequence read archive (SRA) with the SRA accession number SRR17555526 to SRR17555531 and the BioProject accession identity PRJNA795022.

#### Analysis of DEGs

**Figure 4:**
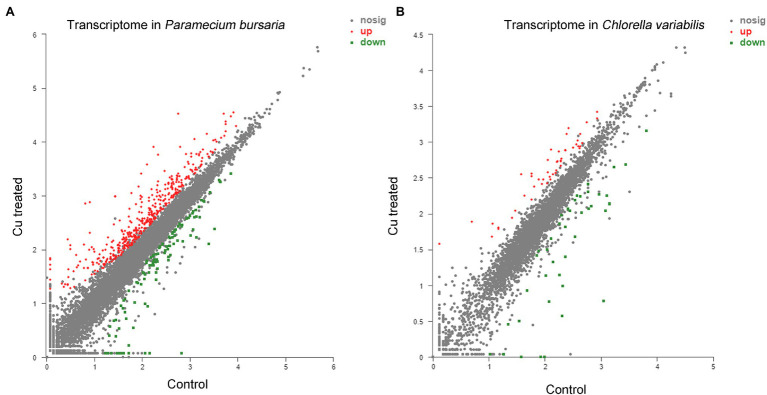
Transcriptome analysis of *P. bursaria* **(A)** and *Chlorella variabilis* **(B)** under control and under CuNP treatment. Each dot represents an individual gene; differentially expressed genes are depicted according to microarray probe signal values. Red dots represent the significantly upregulated genes, and green dots represent the significantly downregulated genes.

Based on those results, a total of 545 DEGs were identified, including 425 upregulated DEGs and 120 downregulated DEGs (Figure 4A), using DESeq2 according to the values of |log_2_ (fold change) | ≥1 and *p*-adjust <0.05, which was achieved by averaging the expression levels of the three replicates. The same parameters were used to screen the DEGs of symbiotic algae *C. variabilis*. Then, 90 DEGs were obtained, 48 of which were upregulated DEGs, and 42 of which were downregulated DEGs (Figure 4B). The highly upregulated and downregulated genes of *P. bursaria* and *C. variabilis* were shown in Supplementary Tables S1–S4. The transcriptome analysis suggesting that the CuNP-treated cells did express genes differently relative to the control.

#### GO and KEGG Enrichment Analysis

To detect the critical functions that were affected by CuNPs, DEGs were mapped to three categories of GO, which included molecular functions (MFs), cellular components (CCs), and biological processes (BPs). GO analysis of *P. bursaria* showed that 14 terms of the top 20 GO enrichment terms were MF, followed by five that were BP, and one that was CC. The major categories that the DEGs affected were ligase activity, forming nitrogen-metal bonds (GO:0051002) and coordination complexes (GO:0051003) and leading to ligase activity, magnesium chelatase activity (GO:0016851), and FMN binding (GO:0010181; Figure 5A). In the top 20 GO enrichment terms of *C. variabilis*, there were 10 BPs and 10 CCs. The DEG principally affected categories were the chlorophyll biosynthetic process (GO: 0015995), tetrapyrrole biosynthetic process (GO:0033014), and porphyrin-containing compound metabolic process (GO:0006778; Figure 5B).

**Figure 5:**
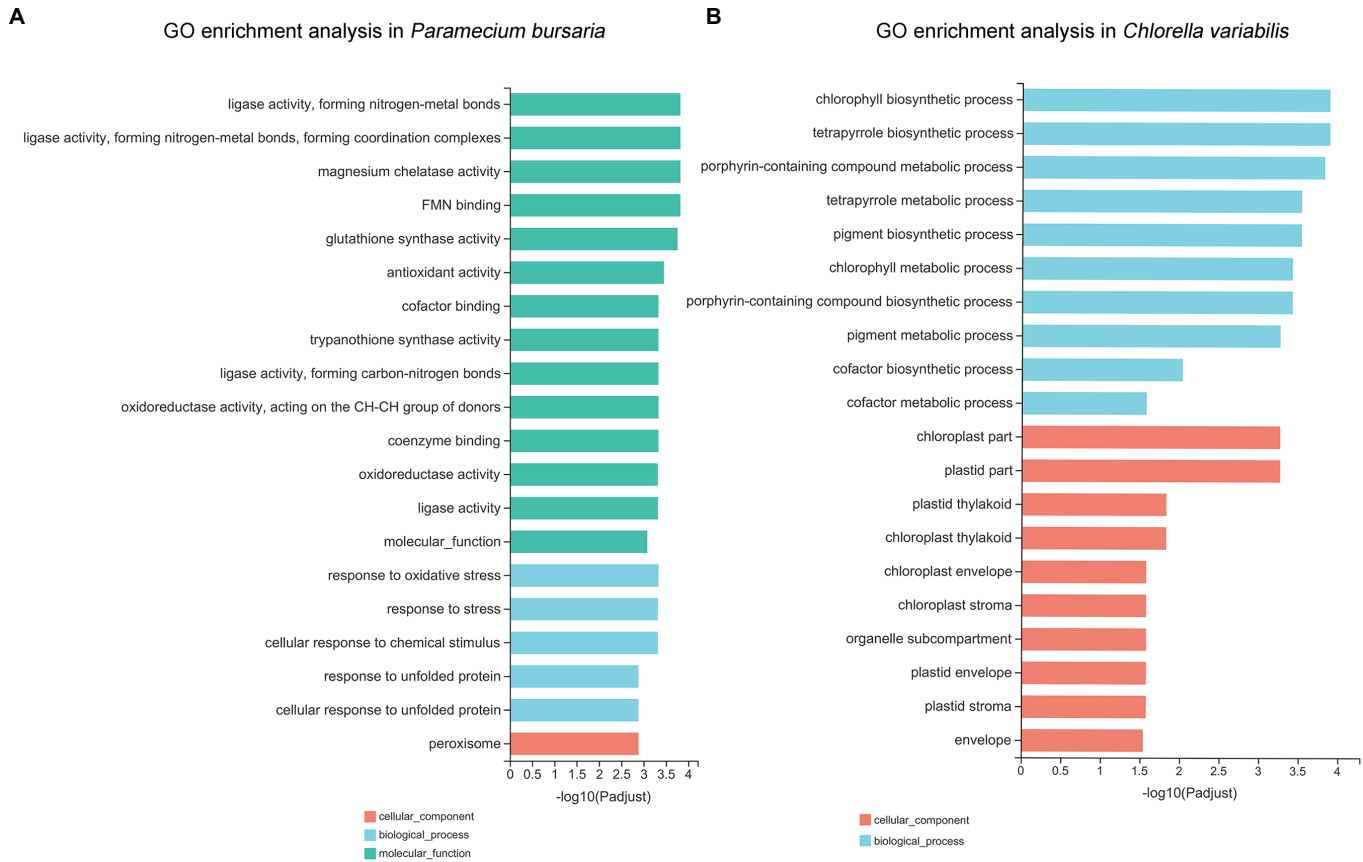
The Gene Ontology (GO) annotations of differentially expressed genes in *P. bursaria* **(A)** and *C. variabilis* **(B)**. The top 20 GO terms identified for biological process, cellular components, and molecular functions are presented.

**Figure 6:**
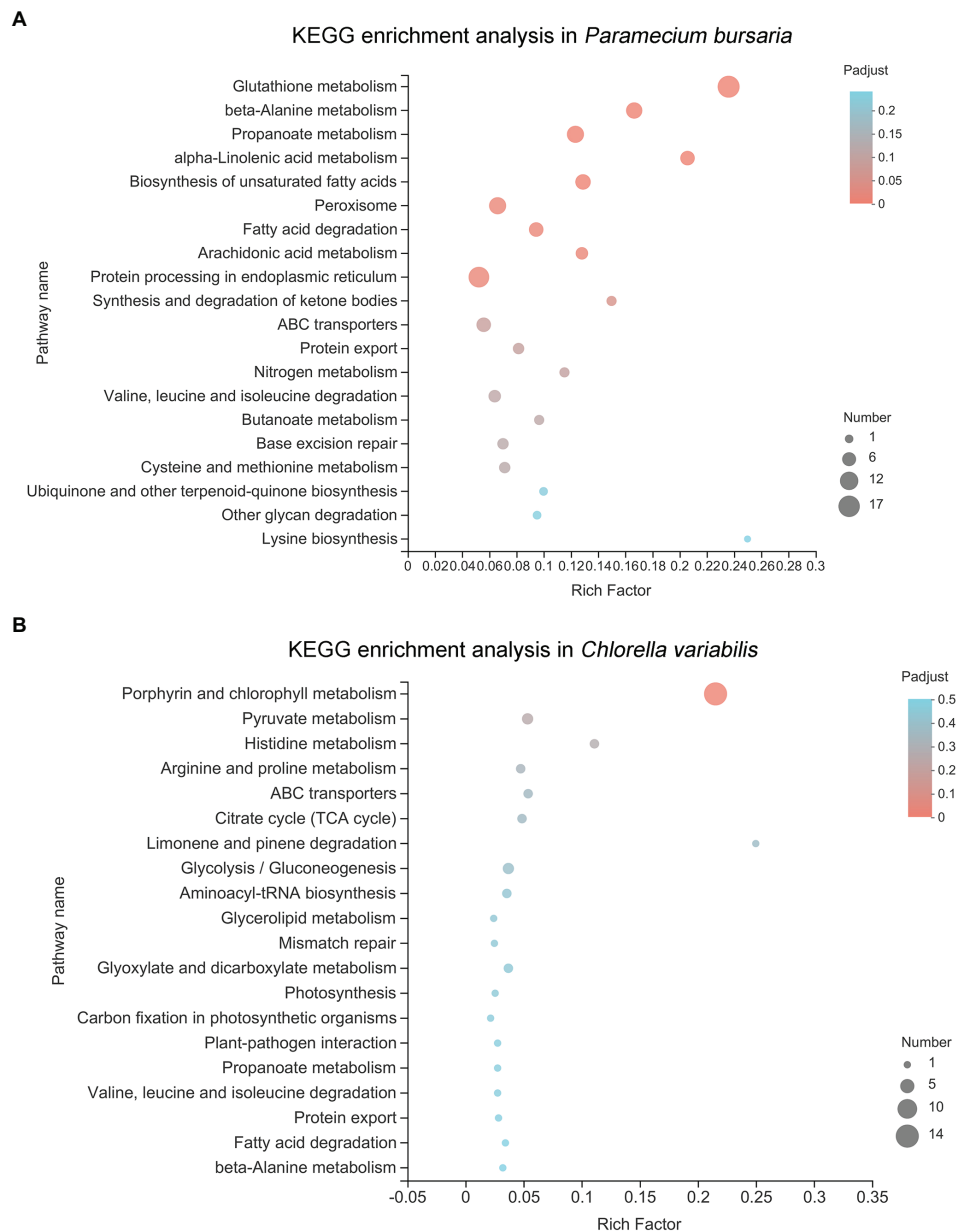
The Kyoto Encyclopedia of Genes and Genomes (KEGG) pathway analysis of differential expressed genes in *P. bursaria* **(A)** and *C. variabilis* **(B)**. The *y*-axis represents pathways and the *x*-axis represents the enrichment score. The color and size of each bubble represent the enrichment significance and the number of genes enriched in the pathway, respectively.

The KEGG ontology was used to better understand which pathways were affected after CuNP exposure. The KEGG ontology showed that the significantly affected pathway in *P. bursaria* was glutathione metabolism, followed by beta-alanine metabolism, propanoate metabolism, alpha-linolenic acid metabolism, and biosynthesis of unsaturated fatty acids (Figure 6A). The symbiotic *C. variabilis* is most significantly enriched in porphyrin and chlorophyll metabolism, followed by pyruvate metabolism, histidine metabolism, arginine and proline metabolism, ABC transporters, and citrate cycle (TCA cycle; Figure 6B). Analysis showed that the KEGG pathways of the host and the symbiosis algae were enriched mainly pointed to cellular oxidative stress and energy metabolism.

#### Validation of RNA Sequencing

To validate the reliability of transcriptome sequencing results in our study, 18 DEGs were randomly selected from *P. bursaria* and *C. variabilis* for further analysis using RT-qPCR. The expression level of these genes using qRT-PCR were in agreement with those using RNA-Seq, which confirmed the effect of CuNPs exposure on the transcriptional response in *P. bursaria* and *C. variabilis* indicated by RNA sequencing (Supplementary Figure S2).

### ROS Production, Enzyme Activity, and Lipid Peroxidation

**Figure 7:**
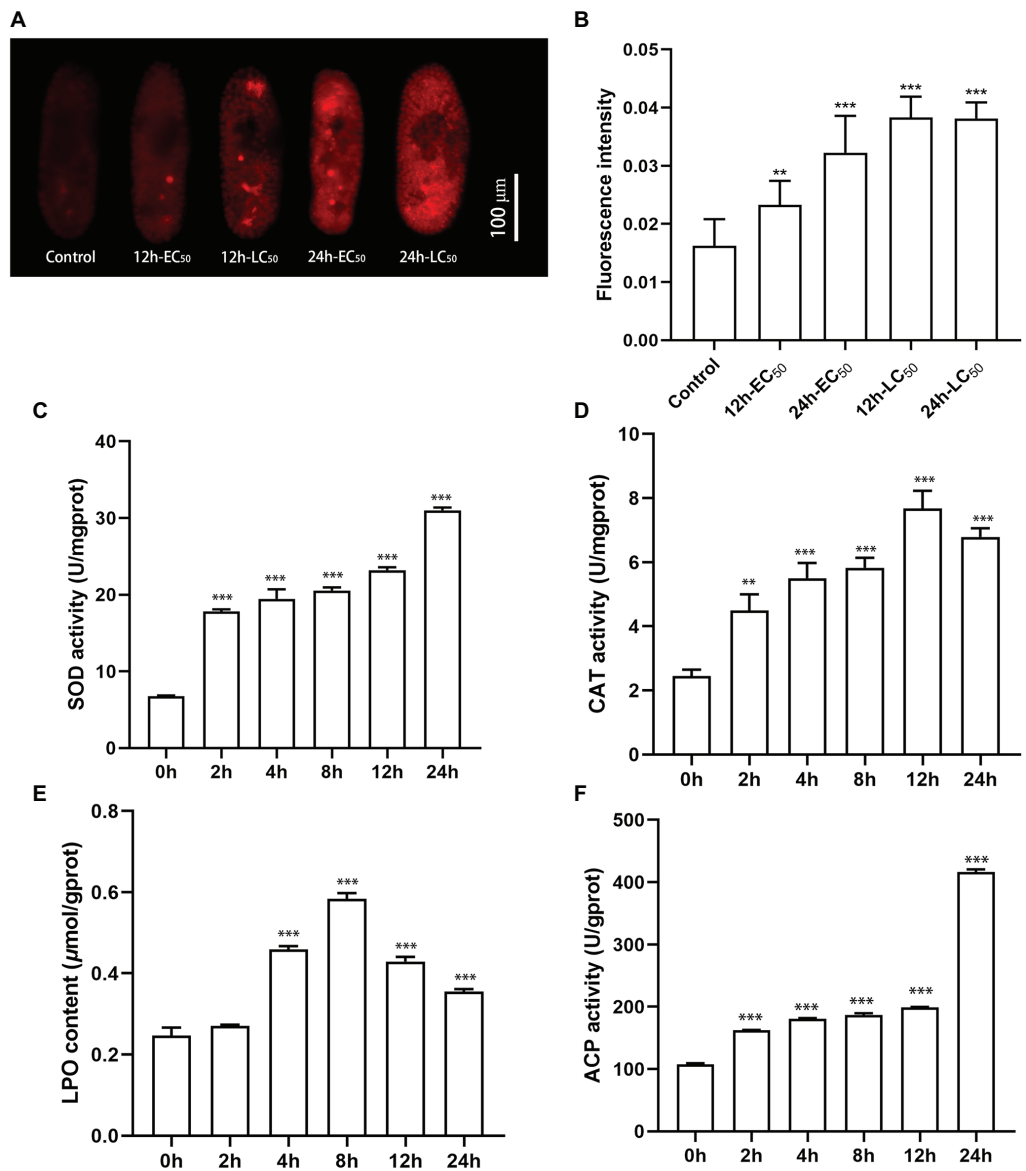
The reactive oxygen species production and enzyme activities of *Paramecium bursaria*–*Chlorella* symbiotic system. **(A,B)** The fluorescence intensity of the DHE probe at different times and different exposure concentrations. **(C–F)** Accumulation of SOD activity, CAT activity, LPO content, and ACP activity under different CuNP exposure times. The statistical comparisons of means with 0 h were performed using one-way ANOVA. Significance levels are as follows: ^**^p < 0.01; ^***^p < 0.001.

The production of ROS in the *P. bursaria*–*Chlorella* symbiotic system induced by CuNPs was measured through a DHE fluorescent probe. Fluorescence graphs show that trace red fluorescence could be observed in the untreated cells, whereas the fluorescence intensity was significantly enhanced after CuNP exposure (Figure 7A). Compared with the control group, the differences of 12 h LC_50_, 24 h LC_50_, and 24 h EC_50_ (*p* < 0.01) were more significant than those of 12 h EC_50_ (*p* < 0.001; Figure 7B). The ROS production was increased with time under low concentration (EC_50_), indicating that the production of ROS is time-dependent in a certain exposure concentration range. The results of comparing the fluorescence intensity of different exposure concentrations with the same exposure time indicated that the production of ROS was in a dose-dependent manner. Fluorescence graphs also show that the fluorescence appeared in both *P. bursaria* cells and symbiotic algae cells, suggesting that both cells in the symbiotic system produced reactive oxygen, and the cells suffered from oxidative stress.

The enzyme activity of SOD and CAT was detected at different exposure durations (Figures 7C,D). We found that the SOD activity increased as the exposure prolonged, whereas the CAT activity increased at the first 12 h and declined slightly at 24 h. These results indicated that exposure to CuNPs caused an increase in the activities of enzymes involved in antioxidants. The LPO content, a measure of the degree of lipid peroxidation, was examined after CuNP exposure. Results showed that CuNPs caused significant lipid peroxidation of the cell membrane, especially at 8 h (Figure 7E). In the present investigation, time-dependent ACP activity was observed in the experiments. A higher level of ACP enzyme activity was observed under CuNP treatments, revealing increases of lysosomes in cells (Figure 7F).

## Discussion

The value of 24 h LC_50_ detected in present study is about 0.05 mg L^−1^, which indicates that *P. bursaria* is more sensitive than most aquatic organisms, such as *Chlorella sorokiniana* (Barreto et al., 2019), *Daphnia pulex* (Garncarek et al., 2019), *Artemia salina* (Cimen et al., 2020), and the barnacle of *Balanus amphitrite* (Yang and Wang, 2019). From the existing data, we suspected that small organisms are generally more susceptible to toxic effects than large organisms (Noureen et al., 2019; Yang and Wang, 2019; Gao et al., 2021; Zhao et al., 2022). The data of NPs concentration in the environment are very few, and the concentration and species of NPs vary greatly in different environments (Bäuerlein et al., 2017). But it can be concluded that the environmental NPs concentrations were mostly lower than the concentrations used in those studies on biological toxicity of NPs (Tuoriniemi et al., 2012; Li et al., 2013; Navratilova et al., 2015; Bäuerlein et al., 2017). Consequently, caution is needed when comparing the results of those studies with the effects of NPs in the environment.

### CuNPs Induced Oxidative Stress and Energy Metabolism Disorder in Both *Paramecium bursaria* and *Chlorella*

Dual RNA-Seq analysis revealed differently expressed genes in both *P. bursaria* and *Chlorella* cells in the CuNP-treated groups compared to the controls. These DEGs were subject to further analysis seeking to understand the molecular pathways and mechanisms that were affected in response to CuNP exposure.

The DEGs of *P. bursaria* were enriched in the KEGG pathway, and many of them were related to oxidative stress, i.e., glutathione metabolism, arachidonic acid metabolism, peroxisome, propanoate metabolism. Antioxidant activity, response to oxidative stress, response to stress, and oxidoreductase activity were also observed in GO terms of the top 20 (Balboa and Balsinde, 2006; Chen et al., 2018). Under normal metabolic conditions, the continuous formation of ROS and other free radicals is important for normal physiological functions, such as the production of ATP, the accompanying cellular redox cycles, and various catabolic and anabolic processes (Rahal et al., 2014). ROS could be balanced by natural antioxidase substances in cells (Gobe and Crane, 2010). The endogenous biological or exogenous environmental factors can cause the excessive generation of free radicals. When the homeostasis process fails and free radicals are generated beyond the capacity of the cell’s defenses, oxidative stress will occur, promoting cellular injury and tissue damage. Oxidative stress has been proved to play an important role in many environmental pollutants or stresses; thus, it should be emphasized in aquatic toxicology and can be considered a potential biomarker for monitoring environmental stress or pollution (Barata et al., 2005; Kim et al., 2010; Becker et al., 2011). Oxidative stress can be induced in organisms after exposure to NPs has been confirmed by many studies (Khan and Shanker, 2015; Suman et al., 2015). In the present study, glutathione metabolism in the KEGG pathway was significantly affected. Glutathione is a tripeptide containing the sulfhydryl group, which has two forms: reduced (G-SH) and oxidized (G-S-S-G; Wilson, 2010). Glutathione peroxidase can catalyze the formation of oxidized glutathione from reduced glutathione while oxidizing reactive oxygen to water; thus, it is an effective antioxidant and plays an important role in oxidative stress (Haenen and Bast, 2014). Catalase is the marker enzyme of peroxisome, which can catalyze the rapid decomposition of hydrogen peroxide to reduce cell damage. The glutathione and peroxisome pathways were enrichment in our transcriptomic date, which indicates that both enzymatic and nonenzymatic antioxidant systems were involved in cellular antioxidant activity. In addition, our results also showed that ROS and lipid peroxides were accumulated in cells, and the activities of related enzymes were significantly increased after CuNP exposure, which further demonstrated that CuNPs disturb the balance between the oxidation and the antioxidation processes. Oxygen stress responses were caused in cells.

Besides, we found that many pathways related to glucose metabolism, amino acid metabolism, and fat metabolism were significantly enriched in *P. bursaria*, including the biosynthesis of unsaturated fatty acids; fatty acid degradation; protein processing in endoplasmic reticulum; valine, leucine, and isoleucine degradation; and other glycan degradation (Mishra et al., 2017). Fatty acids and carbohydrates are the main sources of energy in cells. Branched chain amino acids (valine, leucine, and isoleucine) are important precursors for protein synthesis (Fernstrom, 2005). Their enrichment suggested that proteins were accelerating decomposition to maintain energy homeostasis on intracellular under duress (Hatazawa et al., 2014). A previous study found that the energy-related molecules in the organism are all used up initially under a xenobiotic attack for stress mitigation (Schmidt et al., 2004). Similarly, cells tried to cope with the oxidation stress and repair damaged cell structure after CuNP exposure by producing greater energy-specific molecules and thus replenishing the energy supply. This was achieved by breaking down the energy reserves of the cell (Mishra et al., 2018). Therefore, energy metabolism saw intracellular disorder. Previous studies had shown that toxic effects can lead to glycometabolism disorder, impaired lipid metabolism, and impairment of biological functions associated with amino acids (Chen et al., 2018; Mishra et al., 2018). As mentioned above, the results of this study revealed significant dysregulation of nutrient-related metabolic pathways; energy metabolism in cells was disordered, which may be caused by the increased intracellular energy consumption in response to CuNP-induced stress. Thus, the disturbance of energy metabolism may also be a manifestation of oxidative stress.

Similar to *P. bursaria*, the KEGG pathways and GO terms with *Chlorella* enrichment were also related to oxidative stress (porphyrin and chlorophyll metabolism) and energy metabolism disorder [e.g., pyruvate metabolism, citrate cycle (TCA cycle), glycolysis/gluconeogenesis, propanoate metabolism, fatty acid degradation, plastid part, and plastid thylakoid]. Chu et al. (2015) studied the oxidative stress in *Brassica napus* leaves with chlorophyll deficiency that were associated with porphyrin and chlorophyll metabolism. Plastid is a structure unique to photoautotroph, which is closely related to carbohydrate synthesis and storage (Mauseth, 1998). Most of the pathways related to plastid were upregulated, demonstrating that the production of energy material in symbiotic algae was increased. Pyruvate, an intermediate for the mutual transformation of various substances in cells, is the final product of the glycolysis pathway. After entering mitochondria, pyruvate is oxidized through the TCA cycle to generate energy. As an important metabolic pathway in mitochondria, the TCA cycle is the final metabolic pathway of nutrients (sugars, lipids, and amino acids). The enrichment of the above pathways indicates that oxidative stress can also be induced in the symbiotic algae of *P. bursaria* cells after exposure to CuNPs. Besides, many GO terms in top 20 enrichment in *C. variabilis* were related to chloroplast, and most of them are upregulated, such as the chlorophyll biosynthetic process, porphyrin-containing compound metabolic process, chlorophyll metabolic process, plastid part, and plastid envelope. Cu is one of the transition metals in the electron transport chain of chloroplasts, previous studies demonstrated that Cu activates several enzymes and contributes to RNA synthesis and improves the efficiency of photosystems (Yruela, 2005). These findings indicate that the photosynthesis in algae was enhanced which may be due to the high copper content in the treatment group.

### Cells Were Damaged by ROS Induced by CuNPs

It has been demonstrated that NPs can be internalized in different organisms, whereas the uptake mechanisms and routes have remained unclear (Leclerc and Wilkinson, 2014; Mortimer et al., 2014; Sekine et al., 2017). The most potential mechanism of nanoparticles uptake for animal cells was endocytic pathways, such as phagocytosis, caveolae-mediated endocytosis, and micropinocytosis (Stern et al., 2012; Behra et al., 2013). Unlike the unit membrane in animal cells, the pellicle of protozoan *P. bursaria* is composed of a unit membrane and alveolus and is closely associated with microtubules, cortical mitochondria, trichocyst, and other structures to form the cortex. It seems that nanoparticles cannot easily enter *P. bursaria* through endocytic pathways in the membrane system, and the most likely way is through food vacuoles. This needs to be verified.

The results of the DHE fluorescent probe showed that excessive ROS was produced in cells, which could induce cell damage. This damage may involve the DNA and protein content of the cells with the lipid peroxidation of cellular membranes, calcium influx, mitochondrial swelling, and lysis (Marks et al., 1996). TEM showed significant changes in cell ultrastructure, e.g., mitochondrida and nucleoli. Similar results have been reported in previous studies (Lyu et al., 2019; Zhao et al., 2020). Mitochondria are the “energy factory” of cells, where many important productivity reactions occur, such as oxidative phosphorylation and the tricarboxylic acid cycle. As the most important source of intracellular ROS, mitochondria were most severely damaged during oxidative stress, which is consistent with the TEM images. Extensive damage to mitochondria in cells may lead to an insufficient energy supply and further threaten cell survival. Pan et al. (2021) also found that the toxic of AgNPs likely caused the mitochondrial dysfunction of *T. thermophila*, and the intracellular ATP level was significantly decreased. Nucleoli are the site of ribosome synthesis, which is important for protein translation. Nucleoli will increase in some vigorous metabolism cells for protein synthesis to complete life activities. In the present study, cells were damaged by excessive ROS induced by CuNPs. The process of repairing damaged structures requires the involvement of a large number of new proteins, which may be the cause of nucleolar enlargement. Besides, previous studies had demonstrated that nanoparticles are able to induce DNA damage and some form of mutagenesis (Ng et al., 2010), which may lead to genotoxic effects. Overload of ROS could cause membrane damage, which has been confirmed in bacteria (Das et al., 2015) and human liver cells (Mei et al., 2011). This could also be verified in the results of electron microscopy images and lipid peroxide content determination in the present study.

Higher enzyme activity and lipid peroxidation levels were detected in the present study in cells after CuNP exposure. The increase of SOD activity is directly related to the content of ROS (del Río et al., 2006; Bhaduri and Fulekar, 2012). Superoxide (O_2_^−1^) could be converted into hydrogen peroxide (H_2_O_2_) under higher activity of SOD. Although compared with O_2_^−1^, H_2_O_2_ is less toxic, a higher accumulation of H_2_O_2_ may cause spurred toxic effects on the growth of cells (Melegari et al., 2013; Suman et al., 2015). Thus, the enzyme activity of CAT was upregulated to decompose the excess H_2_O_2_ in CuNP exposure cells. The result of enzyme activity in the present study showed that the activity of CAT was decreased in 24 h, suggesting that the antioxidant system of the cell was not adequate to resist the damage of ROS leading to enzyme destruction. The SOD activity kept rising in 24 h, which led to the massive accumulation of H_2_O_2_, showing a strong toxic effect on cells. Peroxidation of lipid molecules is an indication of oxidative stress; increased LPO content can cause damage to the structure and function of the cell membrane and the organelle membrane. Our data show the content of LPO was accumulated, indicating that the antioxidant system is not adequate to reduce the oxidative stress of the symbiotic system in CuNP treatment, which induced lipid peroxidation.

In conclusion, ROS was produced in cells after exposure to CuNPs, which is detrimental to cells. The antioxidant systems failed to balance the excessive ROS, making the cells suffer oxidative stress and resulting in the damage of cell structure.

### The Balance of the Symbiotic Relationship Was Affected by the Toxicity of CuNPs

The association of *P. bursaria* with symbiotic algae has been regarded as a mutualistic symbiosis. The algae cells provide photosynthetic products to the host, such as oxygen, maltose, and lipid (Reisser, 1976), and the algae are protected by the host from infection with the virus (Kawakami and Kawakami, 1978; Etten et al., 1983). By contrast, the algae cells can be supplied with the nitrogen and carbon sources required for photosynthesis by the host (Albers and Wiessner, 1985). The previous study has demonstrated that the cell length of algae-bearing *P. bursaria* was significantly longer than that of cells without algae and the growth of algae-free *P. bursaria* was significantly inhibited, indicating that the nutrition provided by symbiotic algae is important for the growth of *P. bursaria* (Kodama and Fujishima, 2008). Therefore, the formation of symbiotic relationships improves their survival ability in competition.

Based on previous studies, *P. bursaria* plays a leading role in the symbiotic relationship between *P. bursaria* and *Chlorella*. The *P. bursaria* and symbiotic algae retain the ability to grow independently; the stable endosymbiotic system can be maintained under the condition of benefit to each other. While the balanced symbiotic relationship can be upset under certain environmental conditions. Several studies reported that the number of symbiotic algae could be regulated by the host according to light intensity (Blanc et al., 2010; Kodama et al., 2014; Lowe et al., 2016). Besides, it was demonstrated that the symbiotic algae were eliminated through dark cultivation (Miho and Isoji, 1996; Miwa et al., 1996). The phenomenon of symbiotic algae being digested after *P. bursaria* was stressed has also appeared in previous studies. Lou (2008) reported that the toxicity effect of acephate led to the decrease of symbiotic algae’s number, and there was a significant negative correlation between the concentration of acephate and the number of symbiotic algae. In our study, TEM shows that the symbiotic algae were digested in the host’s DV after *P. bursaria* exposure to CuNPs. The GO term of magnesium chelatase activity was upregulated in RNA-Seq analysis, which is probably due to the decrease of symbiotic algae. Mg^2+^ plays a key role in harvesting solar energy during photosynthesis (Farhat et al., 2014), and it was found that *P. bursaria* may supply its endosymbiotic algae with Mg^2+^ to ensure the algae’s ability to photosynthesize (He et al., 2019). The upregulation of magnesium chelate activity in host cells indicated that the Mg^2+^ provided to the symbiotic algae was reduced, and the symbiotic relationship was affected. Likewise, the enzyme activity of the marker enzyme of the lysosome, ACP, was observed in elevation in the present study, which indicates the increase of autophagy activity or digestive activity in cells. However, numerous DVs were observed under TEM after CuNP treatment for 24 h, indicating that the enhancement of ACP activity may be mainly related to the digestion of the symbiotic system.

The causes for the digestion of the symbiotic algae after *P. bursaria* exposure to CuNPs can be considered as related to the high-energy consumption of cells. In our study, the energy metabolism of *P. bursaria* and *Chlorella* was disordered after exposure to CuNPs. The cells were suffering oxidative stress, and they needed to consume a lot of energy for antioxidant activity, including the production of ATP, structural protein, and antioxidase. The partitioning of energy resources is one of the most difficult tasks an organism has to solve under the influence of stress (Schmidt et al., 2004). Lou (2008) found that the contents of intracellular glucose and maltose were slightly higher than those in the control group at very low concentrations of acephate, whereas with the increase of acephate concentration, the contents of glucose and maltose in *P. bursaria* cells decreased. This indicates that cells will store energy substances after being stressed, and the level of energy consumption will increase with the degree of stress, resulting in a decrease of energy substances stored in cells. Thus, the symbiotic algae suffered from oxidative stress and increased intracellular energy consumption after CuNP treatment, which may reduce the energy supply to host cells.

**Figure 8:**
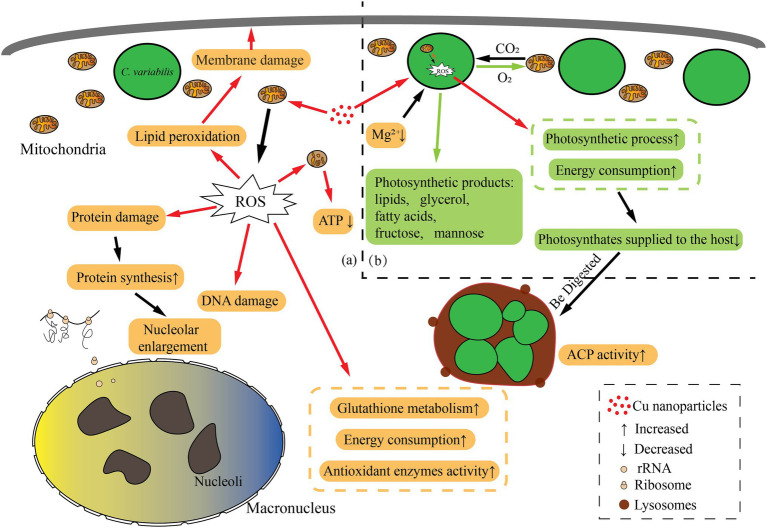
Schematic representation of biological pathways in the *Paramecium bursaria*–*Chlorella* symbiotic system affected by CuNPs. (a) The changed biological pathways in *P. bursaria*; (b) the changed biological pathways in symbiosis algae. The orange box represents the affected pathway within the *P. bursaria*; the green box represents the affected pathway within the *Chlorella*; and the red arrow indicates the damage process.

In conclusion, the *P. bursaria* and symbiotic algae cells suffered oxidative stress, and the increase of intracellular antioxidant activity increased energy consumption. For symbiotic algae, the increase of its energy requirements makes the algae reduce its energy supply to the host, whereas the energy consumption of the host cell also increases. Therefore, some algae were digested to supplement the host’s energy consumption, probably those too damaged to provide energy to their hosts, and the balanced symbiotic relationship was broken (Figure 8). Combined with the previous study, *P. bursaria* may control the number of symbiotic algae according to the cell’s demand for nutrients.

## Data Availability Statement

The data presented in the study are deposited in the SRA repository, accession number PRJNA795022.

## Author Contributions

BN and XF conceived and designed the manuscript. YW and ZG carried out the TEM and SEM research. BT completed the enzyme activity and transcriptomes experiment. BT, XF, and BN wrote the manuscript. All authors contributed to the article and approved the submitted version.

## Funding

This work was supported by the National Natural Science Foundation of China (31672249 and 41876151).

## Conflict of Interest

The authors declare that the research was conducted in the absence of any commercial or financial relationships that could be construed as a potential conflict of interest.

## Publisher’s Note

All claims expressed in this article are solely those of the authors and do not necessarily represent those of their affiliated organizations, or those of the publisher, the editors and the reviewers. Any product that may be evaluated in this article, or claim that may be made by its manufacturer, is not guaranteed or endorsed by the publisher.
