# Supplementary material for: Toxic Effects of Copper Nanoparticles on Paramecium bursaria–Chlorella Symbiotic System
Source: Front Microbiol. 2022 Mar 23;13:834208. doi: 10.3389/fmicb.2022.834208 (PMC8984179; doi:10.3389/fmicb.2022.834208)
Supplement: Supplementary file 1 [file Table_1.DOCX]

**Toxic effects of copper nanoparticles on *Paramecium bursaria*–*Chlorella*** **symbiotic system----** **Supplementary Material**

**Bingyu Tan^1#^, Yiwen Wang^1#^, Zhiwei Gong^2^, Xinpeng Fan^1^, Bing Ni^1*^**

*^1^School of Life Sciences, East China Normal University, Shanghai, China*

*^2^School of Physics and Electronic Sciences, East China Normal University, Shanghai, China*

**#** Bingyu Tan and Yiwen Wang contributed equally to this work.

*Correspondence

E-mail: bni@bio.ecnu.edu.cn

**Table S1.** **The top 20 highly upregulated genes of *P. bursaria*.**

| Gene_id | Gene name | Gene description | FC(CuNPs /Control) | Log_2_FC(CuNPs /Control) | Pvalue | Padjust | Significant | Regulate |
| --- | --- | --- | --- | --- | --- | --- | --- | --- |
| PBUR.KM2.1.G04160057 |  | - | 349.126 | 8.447605 | 2.90242E-11 | 7.74E-09 | yes | up |
| PBUR.KM2.1.G09580040 |  | - | 329.833 | 8.365593 | 8.90405E-11 | 2.18E-08 | yes | up |
| PBUR.KM2.1.G08580069 |  | - | 266.188 | 8.056301 | 5.82653E-09 | 1.01E-06 | yes | up |
| PBUR.KM2.1.G02950023 |  | - | 218.592 | 7.772099 | 5.70942E-09 | 1.00E-06 | yes | up |
| PBUR.KM2.1.G06320099 |  | - | 136.969 | 7.097703 | 2.47386E-07 | 2.94E-05 | yes | up |
| PBUR.KM2.1.G02870013 |  | - | 135.227 | 7.079241 | 0.000561889 | 0.023019 | yes | up |
| PBUR.KM2.1.G01460011 |  | - | 105.37 | 6.719318 | 3.03485E-07 | 3.51E-05 | yes | up |
| PBUR.KM2.1.G09910009 |  | - | 90.32 | 6.496976 | 8.76649E-06 | 0.000664 | yes | up |
| PBUR.KM2.1.G02820042 |  | - | 89.302 | 6.48062 | 5.72607E-18 | 2.87E-15 | yes | up |
| PBUR.KM2.1.G04700012 |  | - | 58.791 | 5.877526 | 1.88454E-25 | 1.78E-22 | yes | up |
| PBUR.KM2.1.G04230001 |  | - | 55.806 | 5.802352 | 3.27908E-17 | 1.49E-14 | yes | up |
| PBUR.KM2.1.G06210017 |  | - | 48.056 | 5.586647 | 2.01947E-12 | 6.35E-10 | yes | up |
| PBUR.KM2.1.G02870014 |  | - | 46.274 | 5.532118 | 1.60345E-39 | 2.81E-36 | yes | up |
| PBUR.KM2.1.G04880004 |  | - | 37.367 | 5.223708 | 5.37968E-41 | 1.10E-37 | yes | up |
| PBUR.KM2.1.G07840004 |  | - | 37.367 | 5.223708 | 5.37968E-41 | 1.10E-37 | yes | up |
| PBUR.KM2.1.G04500005 |  | - | 30.477 | 4.929654 | 1.85866E-40 | 3.51E-37 | yes | up |
| PBUR.KM2.1.G02100005 |  | - | 29.187 | 4.86723 | 3.5412E-19 | 2.17E-16 | yes | up |
| PBUR.KM2.1.G04880003 |  | - | 27.507 | 4.781712 | 1.05044E-67 | 1.29E-63 | yes | up |
| PBUR.KM2.1.G07840003 |  | - | 27.439 | 4.778134 | 6.59264E-84 | 1.62E-79 | yes | up |
| PBUR.KM2.1.G01280030 |  | - | 25.216 | 4.656281 | 1.03002E-12 | 3.37E-10 | yes | up |

**Table S2. The top 20 highly downregulated genes of *P. bursaria*.**

| Gene_id | Gene name | Gene description | FC(CuNPs /Control) | Log_2_FC(CuNPs /Control) | Pvalue | Padjust | Significant | Regulate |
| --- | --- | --- | --- | --- | --- | --- | --- | --- |
| PBUR.KM2.1.G07850018 |  | - | 0.496 | -1.01296 | 1.39E-05 | 0.00101 | yes | down |
| PBUR.KM2.1.G07920069 |  | - | 0.485 | -1.04325 | 0.000631 | 0.025184 | yes | down |
| PBUR.KM2.1.G07950052 |  | - | 0.484 | -1.04795 | 0.001312 | 0.043927 | yes | down |
| PBUR.KM2.1.G03780013 |  | - | 0.484 | -1.04821 | 0.000107 | 0.00587 | yes | down |
| PBUR.KM2.1.G10100013 |  | - | 0.483 | -1.04852 | 5.02E-07 | 5.55E-05 | yes | down |
| PBUR.KM2.1.G02670053 |  | - | 0.476 | -1.07113 | 0.000655 | 0.025969 | yes | down |
| PBUR.KM2.1.G08800033 |  | - | 0.476 | -1.07186 | 3.12E-05 | 0.00206 | yes | down |
| PBUR.KM2.1.G04220012 |  | - | 0.474 | -1.07639 | 2.56E-05 | 0.001725 | yes | down |
| PBUR.KM2.1.G10100014 |  | - | 0.474 | -1.07738 | 0.00019 | 0.009375 | yes | down |
| PBUR.KM2.1.G04410016 |  | - | 0.472 | -1.08198 | 0.000133 | 0.006948 | yes | down |
| PBUR.KM2.1.G10180009 |  | - | 0.472 | -1.08321 | 1.84E-05 | 0.001283 | yes | down |
| PBUR.KM2.1.G04250018 |  | - | 0.472 | -1.08453 | 1.09E-05 | 0.000805 | yes | down |
| PBUR.KM2.1.G08570049 |  | - | 0.471 | -1.08725 | 0.000108 | 0.005901 | yes | down |
| PBUR.KM2.1.G04730019 |  | - | 0.469 | -1.0918 | 5.50E-07 | 5.91E-05 | yes | down |
| PBUR.KM2.1.G05070019 |  | - | 0.469 | -1.0918 | 5.50E-07 | 5.91E-05 | yes | down |
| PBUR.KM2.1.G09600013 |  | - | 0.468 | -1.09497 | 0.000183 | 0.00911 | yes | down |
| PBUR.KM2.1.G02140023 |  | - | 0.466 | -1.10059 | 0.000169 | 0.008597 | yes | down |
| PBUR.KM2.1.G04680021 |  | - | 0.465 | -1.10597 | 0.000184 | 0.00911 | yes | down |
| PBUR.KM2.1.G05210020 |  | - | 0.465 | -1.10597 | 0.000184 | 0.00911 | yes | down |
| PBUR.KM2.1.G01690025 |  | - | 0.456 | -1.13266 | 8.85E-06 | 0.000668 | yes | down |

**Table S3. The top 20 highly upregulated genes of *C. variabilis*.**

| Gene_id | Gene name | Gene description | FC(CuNPs /Control) | Log_2_FC(CuNPs /Control) | Pvalue | Padjust | Significant | Regulate |
| --- | --- | --- | --- | --- | --- | --- | --- | --- |
| gene628 | CHLNCDRAFT_133701 | hypothetical protein | 129.496 | 7.016763 | 1.08E-06 | 0.000397 | yes | up |
| gene3127 | CHLNCDRAFT_143795 | expressed protein | 19.51 | 4.286111 | 1.30E-05 | 0.002803 | yes | up |
| gene3128 | CHLNCDRAFT_143796 | hypothetical protein | 9.657 | 3.271582 | 1.43E-18 | 6.28E-15 | yes | up |
| gene5156 | CHLNCDRAFT_134715 | hypothetical protein | 6.84 | 2.773947 | 7.22E-05 | 0.011291 | yes | up |
| gene3306 | CHLNCDRAFT_144098 | expressed protein | 6.357 | 2.668274 | 4.40E-06 | 0.001293 | yes | up |
| gene7962 | CHLNCDRAFT_14379 | hypothetical protein | 6.173 | 2.625931 | 3.97E-17 | 1.17E-13 | yes | up |
| gene8701 | CHLNCDRAFT_33052 | hypothetical protein | 5.901 | 2.561059 | 1.63E-26 | 1.44E-22 | yes | up |
| gene6270 | CHLNCDRAFT_58394 | hypothetical protein | 5.887 | 2.557459 | 7.83E-06 | 0.00203 | yes | up |
| gene3126 | CHLNCDRAFT_57416 | expressed protein | 5.342 | 2.417279 | 3.40E-06 | 0.001034 | yes | up |
| gene5293 | CHLNCDRAFT_134869 | hypothetical protein | 5.076 | 2.343597 | 2.20E-14 | 4.84E-11 | yes | up |
| gene9738 | CHLNCDRAFT_29092 | hypothetical protein | 4.62 | 2.207798 | 0.000151 | 0.019562 | yes | up |
| gene313 | CHLNCDRAFT_49508 | hypothetical protein | 4.321 | 2.111526 | 0.000249 | 0.028105 | yes | up |
| gene7097 | CHLNCDRAFT_137305 | hypothetical protein | 4.202 | 2.071195 | 2.95E-06 | 0.000963 | yes | up |
| gene2982 | CHLNCDRAFT_34863 | hypothetical protein | 4.106 | 2.037769 | 1.07E-11 | 1.35E-08 | yes | up |
| gene2741 | CHLNCDRAFT_57315 | hypothetical protein | 3.95 | 1.98197 | 1.15E-09 | 9.21E-07 | yes | up |
| gene6941 | CHLNCDRAFT_53914 | hypothetical protein | 3.84 | 1.941239 | 1.78E-07 | 7.85E-05 | yes | up |
| gene2018 | CHLNCDRAFT_142108 | hypothetical protein | 3.783 | 1.919594 | 1.48E-05 | 0.003031 | yes | up |
| gene4128 | CHLNCDRAFT_145238 | hypothetical protein | 3.437 | 1.781118 | 0.000309 | 0.030964 | yes | up |
| gene502 | CHLNCDRAFT_17399 | hypothetical protein | 3.369 | 1.752285 | 2.21E-13 | 3.90E-10 | yes | up |
| gene8134 | CHLNCDRAFT_139411 | hypothetical protein | 3.359 | 1.747831 | 2.01E-08 | 1.18E-05 | yes | up |

**Table S4. The top 20 highly downregulated genes of *C. variabilis*.**

| Gene_id | Gene name | Gene description | FC(CuNPs /Control) | Log_2_FC(CuNPs /Control) | Pvalue | Padjust | Significant | Regulate |
| --- | --- | --- | --- | --- | --- | --- | --- | --- |
| gene8642 | CHLNCDRAFT_59197 | hypothetical protein | 0.481 | -1.055 | 0.000364 | 0.035268 | yes | down |
| gene4018 | CHLNCDRAFT_57665 | hypothetical protein | 0.475 | -1.07394 | 5.98E-05 | 0.010133 | yes | down |
| gene6736 | CHLNCDRAFT_136809 | hypothetical protein | 0.454 | -1.14068 | 0.000278 | 0.029485 | yes | down |
| gene5231 | CHLNCDRAFT_58067 | hypothetical protein | 0.442 | -1.1786 | 0.000326 | 0.032322 | yes | down |
| gene6729 | CHLNCDRAFT_58548 | hypothetical protein | 0.402 | -1.3154 | 6.45E-05 | 0.010647 | yes | down |
| gene6382 | CHLNCDRAFT_58426 | expressed protein | 0.398 | -1.32798 | 5.06E-05 | 0.008922 | yes | down |
| gene1754 | CHLNCDRAFT_50463 | hypothetical protein | 0.36 | -1.47478 | 0.000258 | 0.028439 | yes | down |
| gene4663 | CHLNCDRAFT_134019 | hypothetical protein | 0.342 | -1.54625 | 1.18E-05 | 0.002742 | yes | down |
| gene3604 | CHLNCDRAFT_57538 | expressed protein | 0.281 | -1.82983 | 0.000257 | 0.028439 | yes | down |
| gene390 | CHLNCDRAFT_133364 | hypothetical protein | 0.262 | -1.93462 | 8.19E-06 | 0.002063 | yes | down |
| gene9369 | CHLNCDRAFT_141796 | expressed protein | 0.23 | -2.12105 | 0.000386 | 0.036536 | yes | down |
| gene6944 | CHLNCDRAFT_137099 | hypothetical protein | 0.224 | -2.15739 | 7.36E-12 | 1.08E-08 | yes | down |
| gene832 | CHLNCDRAFT_137777 | hypothetical protein | 0.199 | -2.32592 | 4.72E-05 | 0.008489 | yes | down |
| gene7776 | CHLNCDRAFT_138923 | hypothetical protein | 0.194 | -2.36679 | 8.69E-06 | 0.002128 | yes | down |
| gene2271 | CHLNCDRAFT_142416 | hypothetical protein | 0.188 | -2.41406 | 0.000289 | 0.029819 | yes | down |
| gene9467 | CHLNCDRAFT_142571 | hypothetical protein | 0.178 | -2.49263 | 4.01E-10 | 3.93E-07 | yes | down |
| gene1866 | CHLNCDRAFT_141926 | hypothetical protein | 0.16 | -2.64811 | 2.89E-05 | 0.005529 | yes | down |
| gene6491 | CHLNCDRAFT_53601 | hypothetical protein | 0.146 | -2.77953 | 0.000199 | 0.023997 | yes | down |
| gene355 | CHLNCDRAFT_56532 | hypothetical protein | 0.14 | -2.83591 | 0.000143 | 0.018783 | yes | down |
| gene4922 | CHLNCDRAFT_48847 | hypothetical protein | 0.135 | -2.89214 | 8.93E-05 | 0.013344 | yes | down |


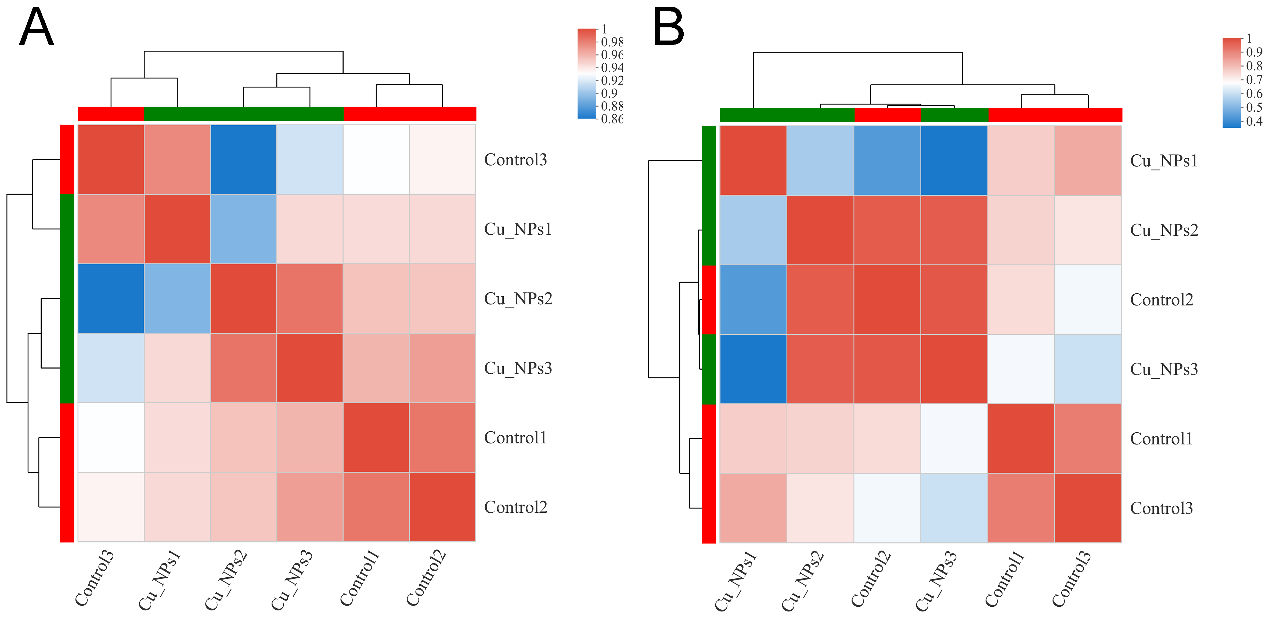


**FIGURE S1.** The correlation analysis of the overall transcription expression of *Paramecium bursaria* **(A)** and *Chlorella variabilis* **(B)**.


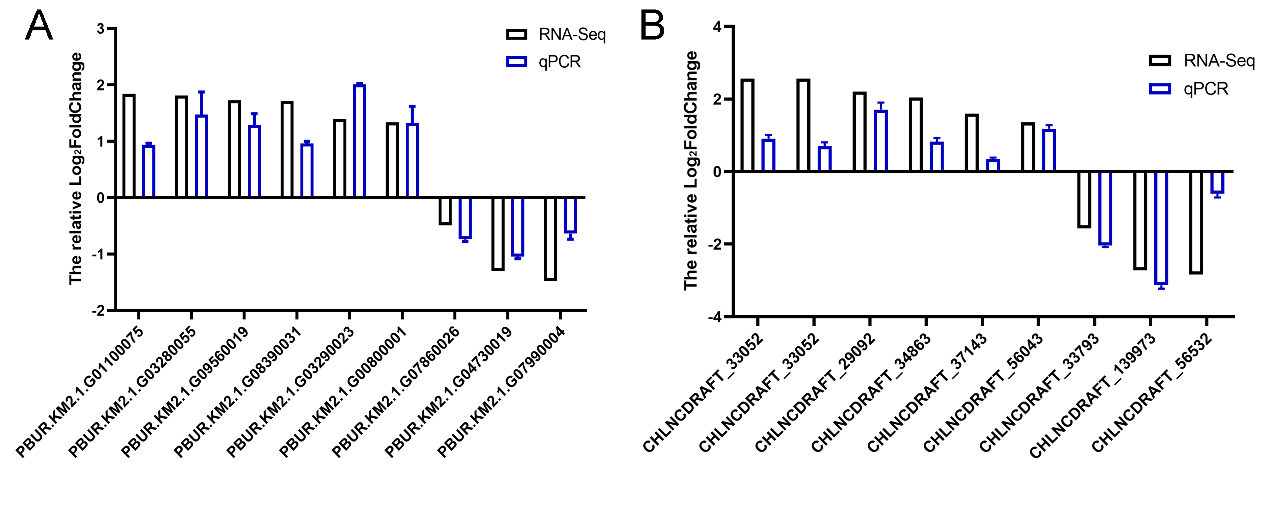


**FIGURE S2.** Validation of RNA-Seq profiles using real-time qPCR, *Paramecium bursaria* **(A)** and *Chlorella variabilis* **(B)**.
